# Novel sedentary cage induced sedentariness in rats: evidence from relevant biomarkers

**DOI:** 10.1186/s12902-022-01221-1

**Published:** 2022-11-25

**Authors:** Quadri K. Alabi, Rufus O. Akomolafe

**Affiliations:** 1 Department of Physiology, Faculty of Basic Medical Sciences, Adeleke University, Ede, Osun State Nigeria; 2 Department of Physiological Sciences, Faculty of Basic Medical Sciences, Obafemi Awolowo University, Ile-Ife, Osun State Nigeria

**Keywords:** Sedentary behavior, Sedentary cage, Anthropometric indices, Insulin, Blood pressure, cardiometabolic syndromes

## Abstract

**Background:**

Sedentary behavior or physical inactivity is considered a foremost contributor to the rise in obesity and overweight and a risk factor for several non-communicable diseases. However, its effect on the etiopathogenesis of some diseases is underestimated in both developed and developing countries worldwide. The present study designed a novel sedentary cage with a view to achieving sedentariness in rats, and also investigated the effectiveness of the cage in achieving sedentariness by assessing some markers of cardiometabolic risks in Wistar rats.

**Methods:**

Adult male Wistar rats were divided into 3 groups of six rats. Rats in Group 1 were the control. The sedentary groups were 4-hr. sedentary and 8-hr. sedentary. The sedentary rats were subjected to restrained movements for 4 and 8 hours daily in the sedentary cage for 3 months. Anthropometric indices, food consumption and blood pressure parameters of the rats were measured. Microalbuminuria and serum glucose, uric acid, albumin, nitric oxide, endothelin-1, insulin, inflammatory markers were also Measured.

**Results:**

Results indicated significant increases in body weight, BMI, Lee index, food consumption, systolic and diastolic pressure and decrease in serum nitric oxide bioavailability in the 8-hr sedentary rats. There were also significant increases in serum glucose, uric acid, endothelin-1, insulin, CRP and microalbuminuria in the 8-hr. sedentary rats in comparison with the control. The interleukin-6 and TNF-α also revealed a significant increase in the 8-hr. sedentary rats compared with the control. However, there was no significant difference in cortisol level across all the groups.

**Conclusions:**

We concluded that the novel sedentary cage successfully caused sedentariness in the rats as evident by the alteration in the cardiometabolic health in the rats, especially the group that were made sedentary for 8 h.

## Background

Physical inactivity is considered a foremost contributor to the rise in obesity and overweight and a risk factor for several non-communicable diseases such as diabetes mellitus, cardiovascular disease, breast and colon cancers [1–4]. It also contributes to compromised mental health [5], hasten the onset of dementia [6], causing abnormal weight gain [1] and global mortality [3]. However, physical activity has helped for mental health stability [5], contributed to healthy weight [1], and general wellbeing [7].

Sedentary behavior (SB) is any waking behavior while in a sitting, resting, or lying position with little energy expenditure [8]. Recent studies have indicated that prolonged sedentary behaviors are linked with metabolic disorders, cardiovascular disease, and type 2 diabetes (T2DM) as well as mortality [9, 10].

The Prevalence of SB is regarded as a global public health problem owing to recent technological innovations and the changeover towards more sedentary occupations and entertainment such as television and phone viewing, games, social media and the likes. The use of personal motorized/commercial transportation also contributes to swiftly changing patterns of individual activity from active position to sedentary position across the globe.

According to the World Health Organization (WHO), current global estimates of physical inactivity show 27.5% of adults [11] and 81% of adolescents [12], who are not sufficiently active [13] and there has been no favorable improvement in these estimations over the last decade. The prevalence of insufficient activity of individuals is due to the increasing westernisation, urbanisation and mechanisation occurring in most countries around the world. This has resulted in a global rise of several non-communicable diseases with more than 5 million deaths recorded globally each year [2]. Although several human studies have emerged on health complication relating to SB [14, 15], a validated animal model for SB and its associated health complications are still a subject of study.

**Fig. 1:**
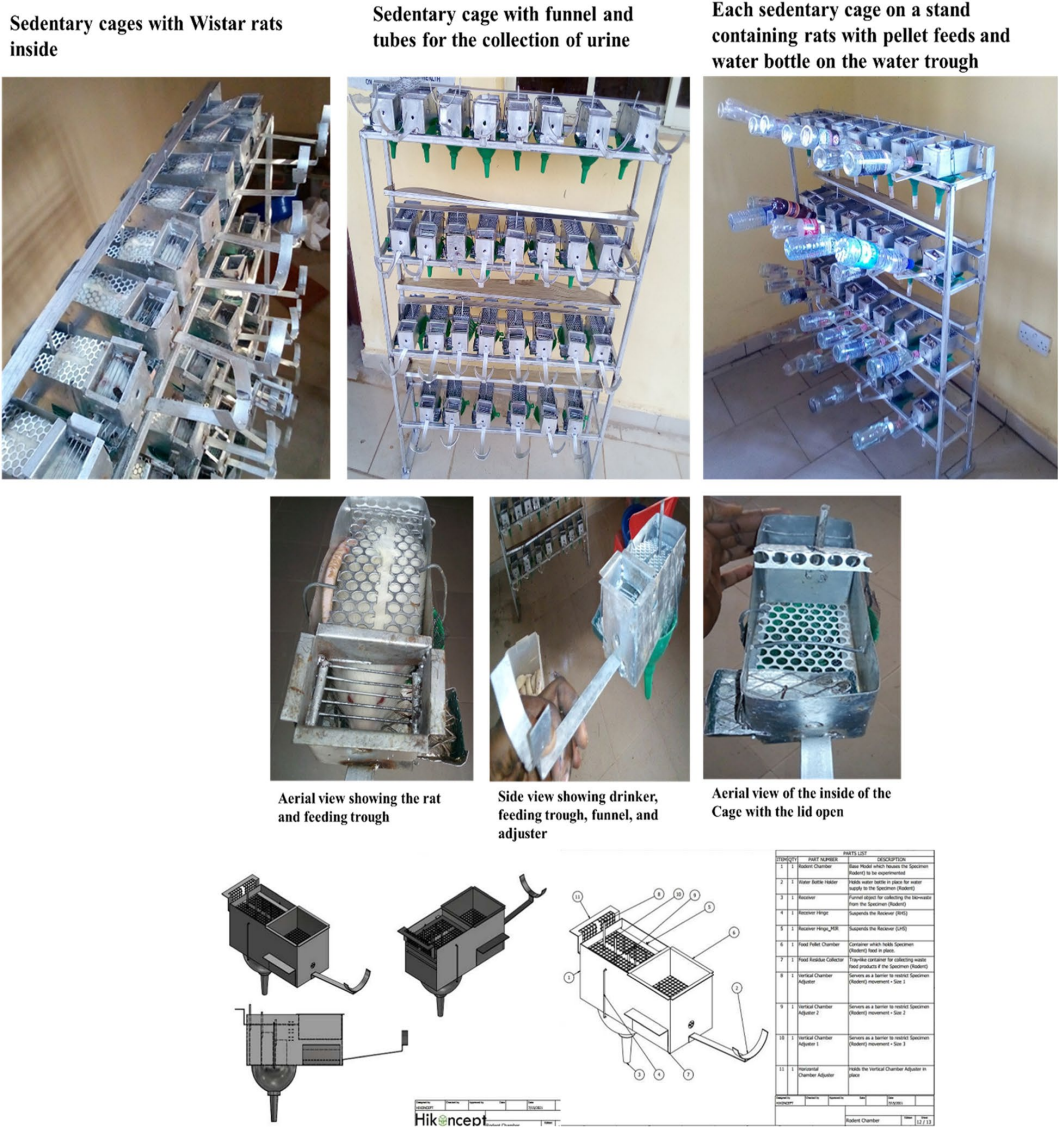
Sedentary Cage

Most of the animal models of sedentariness used by previous researchers inflicted some other stressors apart from the restriction of movement they were meant to achieve. The present study designed a novel sedentary cage that attempted to eliminate the other stressors that were associated with the previous ones. The cage (Fig. 1) was specially made to ensure the restriction of Wistar rats in a way that mimicked the sedentary behavioral patterns that were peculiar to humans. This cage consisted of some features that made it quite different from previous models of sedentary behaviors in laboratory animals. It was made of iron steel having a housing compartment of 20 cm length, 9 cm breadth and 10 cm height, in which the rat were separately kept during sedentary period. It has adjustable shutters which can be easily moved both vertically and horizontally as the rat increased in size due to growth. It also had feeding and water troughs where feeds and water were kept, and a funnel attached to the bottom of each cage that can be used to collect neat samples of the urine of the rats. The cage was used to restrict the movement of the rats for certain number of hours to mimic the sedentary lifestyle associated with humans without subjecting the rats to other stressors, apart from restriction of movement. The cage was firmly held on a specially constructed wooden stand to enable easy collection of urine sample from the rats. This cage was suitable for both juvenile and adult rat model of sedentary behavior, unlike rodent wheel lock that was mostly used for juvenile rats. Housing rats inside the cages was able to restrict their movement as much as possible without inflicting any pain, because of all the unique features of the cage. Therefore, the model paralleled what usually occurs in current-day adolescents regarding the mechanistic initiation of risk factors associated with sedentariness.

The cage was designed to deny the rats access to physical activity for certain period of time in animals and it provided a physiological model of physical inactivity that differs from the wheel lock and other drastic models of sedentariness such as tail suspension, hindlimb unloading and hindlimb immobilization, which inflicted postural alteration, inflammation and pain to the hindlimb and tail of the animals respectively. The novel sedenatary cage prevented physical activity in animals without a drastic alteration of their postures and also provided a unique device to study human-relevant translational model of development of complex diseases associated with physical inactivity.

Owing to the increase in recent technological innovations around the world, individuals in many developed and developing countries are facing challenges related to a sedentary lifestyle, which happens to be the etiology of some chronic diseases [16–20]. Although, studies have reported the health risks of sedentary behavior, many of these studies were population-based cross-sectional or longitudinal studies [18–22], with very few findings from animal models of sedentariness [23, 24].

With the high prevalence and detrimental effects of SB on health, changing sedentary behavioral patterns within a population is a major public health concern. To develop interventions that decrease sedentary time, a better understanding of its underlying determinants is needed. Research on the animal model for SB and its health complications are of utmost importance.

The present study designed a novel sedentary cage with a view to achieving sedentariness in rats, and also investigated the effectiveness of the cage in achieving sedentariness by assessing some biomarkers of cardiometabolic health in Wistar rats subjected to two different periods of sedentariness in the cage for a period of 3 months.

## Materials and methods

### Materials

The novel sedentary cage was designed by the researchers and constructed in conjunction with the Central Technological Laboratory and Workshops, Obafemi Awolowo University (OAU), Ile-Ife, Nigeria.

### Animal care and management

Eighteen (18) adults male Wistar rats weighing 100 g - 113 g (4–5 weeks of age) were used for this study. The rats were procured from the Animal House of the College of Health Sciences, OAU, Ile-Ife. They were housed in the same laboratory where the study was carried out at room temperature of about 30–32 °C. They were allowed to have free access to standard rat pellet chow (Table 1) (CAP Feed LTD. Oshogbo, Nigeria) and water ad libitum. The experiment was approved by the Health Research Ethics Committee (HREC), Institutes of Public Health, Obafemi Awolowo University (OAU), Ile-Ife, and in accordance with the National Academy of Sciences Guide for the Care and Use of Laboratory Animals [25]. The study was also carried out in line with the ARRIVE guidelines for animals in research (reporting in vivo experiment) [26].

**Table 1:** Normal Feed Formulation

Ingredients	Unit	Quantity	Amount expressed in percentage (%)
Maize White	G	12	20
Wheat Bran	G	39	10
Palm Kernel Cake-Local	G	27	12
Groundnut Cake	G	27	12
Soya Bean Meal	G	12	12.6
Rice Bran	G	27.75	11
Lysine	G	0.48	1.0
Methionine	G	0.33	1.0
Tryptophan	G	0.12	0.4
Premix Broiler	G	0.41	0.6
Bone Meal	G	1.50	2.0
Limestone	G	1.53	0.6
Common Salt	Μg	0.03	0.2
Calcium phosphate	G	0.25	1.0
Potassium citrate	G	0.12	1.0
Iron (mg)	Mg	1.00	1.2
Casein, vitamin tested	G	10.5	5.6
Folic acid	Mg	0.3	0.5
B_12_	Mg	10.4	4.6

### Experimental design

The rats were randomly divided into three groups with each group consisting of six rats.

#### Calculation of sample size and justification

The resource equation method [27, 28] of animal sample size was adopted in this study;

E = Total number of animals – Total number of groups.

E = (Total number of animals x Total number of groups) – Total number of groups.

E is measured as the degree of freedom of analysis of variance (ANOVA) and the adequate value of E lies between 10 and 20. Less than 10 will decrease the chance of getting significant result while more than 20 will result to wastage of animals and resource.

E = (6 × 3) – 3.

E = 18–3 = 15.

E = 15 this is considered as adequate sample size because the value was within acceptable limit.

Group 1 (the control or non-sedentary): The rats were kept in the conventional rat cage to have free movement and other activities like access to water and food ad libitum for 3 months.

Group 2 (4 h sedentary group): The rats were kept singly in the novel sedentary cage for 4 hrs. (8 am – 12 noon) daily for 3 months.

Group 3 (8 h sedentary group): The rats were kept singly in the novel sedentary cage for 8 hrs. (8 am – 4 pm) daily for 3 months.

During the period of restriction, each rat from groups 2 and 3 had unrestricted access to food and water in the novel sedentary cage throughout the study. The Wistar rats in groups 2 and 3 were kept in sedentary cages for 4 hours and 8 hours daily and respectively. They were transferred to convectional cages after the hours of sedentariness elapsed to allow free movement.

### Measurement of body weights and food consumption

The body weights of the rats were measured once every week with the aid of a digital weighing balance (Kerro, China) and their weight gain or loss was calculated. Their food consumption while inside the sedentary cage was measured daily at the end of the period of sedentariness.

The percentage weight gain was then calculated using the formulae below;

$$\textrm{Body}\ \textrm{Weight}\ \textrm{Change}\ \left(\textrm{BWC}\right)\%=\frac{\left(\textrm{Final}\ \textrm{body}\ \textrm{weight}-\textrm{Initial}\ \textrm{body}\ \textrm{weight}\right)\ \textrm{g}}{\textrm{Initial}\ \textrm{body}\ \textrm{weight}\ \left(\textrm{g}\right)}\times 100$$

### The anthropometric indices

The anthropometric indices of each rat were determined once a week.

The body length of the rats was measure from the head (nose region) to anus, using the tape rule.

The results of the body weight and length were used to determine the following anthropometric indices:

1. Body mass index (BMI) = body weight (g)/length^2^ (cm^2^)
2. Lee index = cube root of body weight (g) / nose-to-anus length (cm) [29].

### Blood pressure measurement

After the 3 months of study, the blood pressures of both the non-sedentary and sedentary rats were taken using an automated noninvasive blood pressure system (CODA™ mouse rat tail-cuff system, Kent Scientific Corporation, Torrington, Connecticut, USA). Other blood pressure parameters such as systolic blood pressure, diastolic blood pressure and mean arterial blood pressure were assessed noninvasively in the rats through tail plethysmography. The measurements were carried out in triplicate.

### Blood collection

Twenty-four hours after the last day of the experiment, all the rats were sacrificed under ketamine hydrochloride anesthesia (60 mg/kg of body weight via intramuscular route). The blood sample (about 4–5 ml) of each rat was collected by cardiac puncture into a separate plain tube and centrifuged at 4000 revolution per minute for 15 minutes at 4 °C, using a centrifuge (Centurium Scientific, Model 8881) to separate the serum (about 55% of the total blood volume). The serum was collected into separate plain tube for biochemical analysis.

### Biochemical assays

#### Assays of serum uric acid, glucose, albumin, nitric oxide and lipid profile

The serum samples were analyzed for uric acid, glucose, albumin, nitric oxide and total cholesterol (TC), triglycerides (TG), and high-density lipoprotein (HDL) concentrations measured using the kits purchased from Randox laboratories Ltd. (Crumlin, Co. Antrim, United Kingdom). Also, urinary albumin, creatinine and albumin-creatinine ratio were assessed by using Randox laboratory kits and calculated to obtain microalbuminuria or albuminuria.

##### Low density lipoprotein–cholesterol (LDL-c) in the serum

This was determined using Friedewald et al., [30] equation below;

LDL-_C_ conc. = Total Cholesterol – (Triglyceride/5) – HDL-_C_.

The concentration of LDL-_C_ was expressed in mg/dl.

##### Atherogenic index (AI)

Atherogenic index was determined using the equation below;

AI = Log_10_ (TG/HDL-_C_) [31].

#### Assays of serum insulin, cortisol and C-reactive protein (CRP)

Serum endothelin-1 (CAT. NO.: ab133030) and CRP (CAT. NO.: ab108827) were determined using specific rat ELISA kits purchased from Abcam (USA). Cortisol (CSB-E05112r) was estimated using rats ELISA kit from Cusabio Biotech Co., Ltd. (Wuhan, China). Interleukin (IL)–6 and TNF-α were using the ELISA kits procured from Wkea Med Supplies Corp (Changchun, Jilin, China).

#### Assessment of insulin and insulin resistance

Serum insulin was determined using rat ELISA kits obtained from Elabscience (CAT. NO.: E-EL-R3034; Wuhan, China). Homeostatic Model Assessment of Insulin Resistance (HOMA-IR) was estimated by using the following formula:

Fasting blood glucose (mg/dL) × Fasting serum insulin (uIU/mL) /22.5 [32]. Low HOMA-IR values indicate high insulin sensitivity, while high HOMA-IR values indicate insulin resistance (low insulin sensitivity).

### Statistical analysis

All data were expressed as means ± standard deviation (SD). The statistical analysis was performed using one-way analysis of variance (ANOVA) followed by Neumann Keul’s post hoc test for comparison between groups. Correlation between the variables was determined. Differences were considered significant when *p* <  0.05 (Graph Pad Software Inc., San Diego, CA, USA).

## Results

### Anthropometric indices and food consumption

The initial body weight of all the rats showed no significant difference (F = 0.09; *p* = 0.91) at the beginning of the study (Table 2).

**Table 2:** Effect of 3 months sedentariness on anthropometric indices, food consumption and blood pressure of the rats

Parameters/Groups	Control	4 h sedentary	8 h sedentary
**Initial Body Weight (g)**	112.3 ± **6.59**	112.8 ± **6.62**	113.0 ± **7.15**
**Final Body Weight (g)**	209.7 ± 12.99	222.2 ± 18.04	287.5 ± 18.63*^#^
**Body Weight Changes (%)**	86.83 ± 8.38	96.17 ± 12.43	152.5 ± 13.29*^#^
**Body Length (cm)**	19.12 ± 0.29	18.25 ± 0.28*	18.38 ± 0.44*
**BMI (g/m**^**2**^**)**	0.57 ± 0.03	0.67 ± 0.28*	0.83 ± 0.02*^#^
**Lee Index**	0.31 ± 0.004	0.33 ± 0.008*	0.36 ± 0.005*^#^
**Food Consumption (g)**	16.67 ± 2.66	28.17 ± 6.49*	37.17 ± 3.76*^#^
**Systolic Blood Pressure (mmHg)**	122.5 ± 23.36	140.2 ± 17.38	180.8 ± 30.29*^#^
**Diastolic Blood Pressure (mmHg)**	100.5 ± 12.10	117.2 ± 5.89	156.3 ± 13.26*^#^
**Mean Arterial Pressure (mmHg)**	107.9 ± 13.61	136.1 ± 32.61*	164.4 ± 17.70*^#^

Each value represents Mean ± SD (*n* = 6)

* = significantly different from control (*p* < 0.05);

# = significantly different from 4 h. SED (*p* < 0.05)

At the end of the study, both the final body weight and weight gain of the rats in 8 h. sedentary was significantly increased (F = 37.39; *p* <  0.0001; F = 56.59; *p* <  0.0001 respectively) when compared to the control and 4 h. sedentary rats (Table 2).

A significantly shorter (F = 5.142; *p* = 0.0199) body length was noted in 8 h. sedentary group when compared with the control and 4 h. sedentary groups (Table 2).

The body mass index (BMI) of 4 h. sedentary and 8 h. sedentary groups were significantly higher (F = 135.6; *p* <  0.0001) when compared with the control group. Also, a significantly higher BMI (t = 12.15; *p* < 0.0001) was recorded in the 8 h. sedentary group when compared with the 4 h. sedentary group (Table 2).

The Lee index of the rats in the 4 and 8 h. sedentary groups were significantly higher (F = 93.17; *p* < 0.0001) when compared with the control. Similarly, there was a significantly higher Lee index in rats in the 8 h. sedentary group (t = 7.60; *p* < 0.0001) when compared with the 4 h. sedentary group (Table 2).

The food consumption of the 4 and 8 h. sedentary groups were significantly higher (F = 29.98; *p* < 0.0001) when compared with the control. Also, the 8 h. sedentary group had a significantly higher (t = 6.53; *p* = 0.00286) food consumption when compared with 4 h. sedentary (Table 2).

### Blood pressure parameters

The systolic blood pressure, diastolic blood pressure and mean arterial pressure were significantly higher (F = 9.115; *p* = 0.0026; F = 10.46; *p* = 0.0014 and F = 9.187; *p* = 0.0025 respectively) in the 8-hr. sedentary group when compared with the control and even the 4-hr sedentary group (Table 2). Thus, the significantly higher values of blood pressure parameters in the 8 hr. sedentary group were an indication of hypertension and vascular dysfunction (Table. 2).

### Lipid profile

Total cholesterol (TC) of the 4 and 8 h. sedentary groups were significantly higher (F = 48.67; *p* < 0.0001) when compared with the control. Also, TC of the rats in the 8 h. sedentary group was significantly higher (t = 5.85; *p* = 0.0002) when compared with the 4 h. sedentary group (Table 3). The serum total triglycerides (TG) of the 4 and 8 h. sedentary groups were significantly higher (F = 55.92; *p* < 0.0001) when compared with the control. Also, the TG of rats in the 8 h. sedentary group was significantly higher (t = 6.22; *p* < 0.0001) when compared with the 4 h. sedentary group (Table 3). Low density lipoprotein (LDL) of rats in the 4 and 8 h. sedentary were significantly higher (F = 23.43; *p* < 0.0001) when compared with the control. Also, the 8 h. sedentary group had a significantly higher LDL (t = 3.55; *p* = 0.0053) when compared with the 4 h. sedentary group (Table 3). The high density lipoprotein (HDL) of the rats in the 8 h. sedentary group was significantly higher (F = 7.104; *p* = 0.0068) when compared with the control and 4 h. sedentary groups (Table 3). Atherogenic Index (AI) of the 4 and 8 h. sedentary groups were significantly higher (F = 56.90; *p* < 0.0001) when compared with the control. Also, the 8 h. sedentary group had a significantly higher AI (t = 6.68; *p* < 0.0001) when compared with 4 h. sedentary group (Table 3).

**Table 3:** Effect of 3 months sedentariness on lipid profile of the rats

Parameters (Serum) /Groups	Control	4 h sedentary	8 h sedentary
**Total Cholesterol (mg/dl)**	65.19 ± 7.46	87.77 ± 8.30*	143.5 ± 21.81*^#^
**Triglyceride (mg/dl)**	61.05 ± 9.42	86.14 ± 8.81*	132.9 ± 16.19*^#^
**Low Density Lipoprotein (mg/dl)**	41.99 ± 12.11	74.16 ± 14.38*	122.5 ± 30.14*^#^
**High Density Lipoprotein (mg/dl)**	35.45 ± 6.98	33.06 ± 5.45	23.82 ± 4.15*^#^
**Atherogenic Index**	0.24 ± 0.08	0.42 ± 0.10*	0.75 ± 0.08*^#^

Each value represents Mean ± SD (*n* = 6)

* = significantly different from control (*p* < 0.05);

# = significantly different from 4 h. SED (*p* < 0.05)

### Serum uric acid, glucose, albumin and nitric oxide

Serum uric acid of rats in the 8 h. sedentary group was significantly higher (F = 5.351; *p* = 0.0176) when compared with the control and 4 h. sedentary groups (Table 4). There was a significantly higher (F = 92.98; *p* < 0.0001) serum glucose concentration in rats in the 8 h. sedentary group when compared with the control and 4 h. sedentary groups (Table 4). Serum albumin of rats in the 8 h. sedentary was significantly lower (F = 8.920; *p* = 0.0028) when compared with the control and 4 h. sedentary group (Table 4). Similarly, serum nitric oxide was significantly lower (F = 16.39; *p* = 0.0002) in the 8 h. sedentary group when compared with the other groups (Table 4).

**Table 4:** Effect of 3 months sedentariness on serum uric acid, glucose, albumin and nitric oxide and urinary albumin and creatinine and Albumin-creatinine ratio of Rats

Groups/Parameters	Control	4 h sedentary	8 h sedentary
**Serum Uric Acid (mg/dl)**	5.65 ± 1.10	6.39 ± 0.66	8.07 ± 1.88*^#^
**Serum Glucose (mg/dl)**	91.60 ± 10.15	106.2 ± 13.79	189.6 ± 15.77*^#^
**Serum Albumin (mg/dl)**	4.78 ± 0.78	5.36 ± 1.03	3.26 ± 0.83*^#^
**Serum NO (mmol nitrite/mg protein)**	2.33 ± 0.57	1.90 ± 0.40	0.97 ± 0.20*^#^
**Urinary creatinine (mg/dl)**	47.68 ± 7.78	41.83 ± 8.32	24.90 ± 6.42*^#^
**Urinary albumin (mg/dl)**	0.40 ± 0.14	0.51 ± 0.16	0.81 ± 0.15*^#^
**Albumin-to-Creatinine ratio (mg/g)**	8.81 ± 3.49	13.33 ± 7.85	34.00 ± 9.15*^#^

Each value represents Mean ± SD (*n* = 6)

* = significantly different from control (*p* < 0.05);

α = significantly different from 4 h. SED (*p* < 0.05)

### Urine albumin, creatinine and albumin-to-creatinine ratio

There was an indication of significant microalbuminuria in the 8-h. sedentary group when compared to with the control and 4-hr sedentary groups. Urine albumin and creatinine were significantly higher (F = 11.33; *p* = 0.0010; F = 14.74; *p* = 0.0003 respectively) in the 8 h. sedentary group when compared with the control and 4-h. sedentary groups. Also, the urine Albumin-to Creatinine ratio was significantly higher (F = 20.62; *p* < 0.0001) in the 8-h. sedentary group when compared with the control and 4 h. sedentary groups, indicating occurrence of microalbuminuria in this group (Table 4).

### Serum cortisol, insulin and insulin resistance (HOMA-IR)

**Fig. 2:**
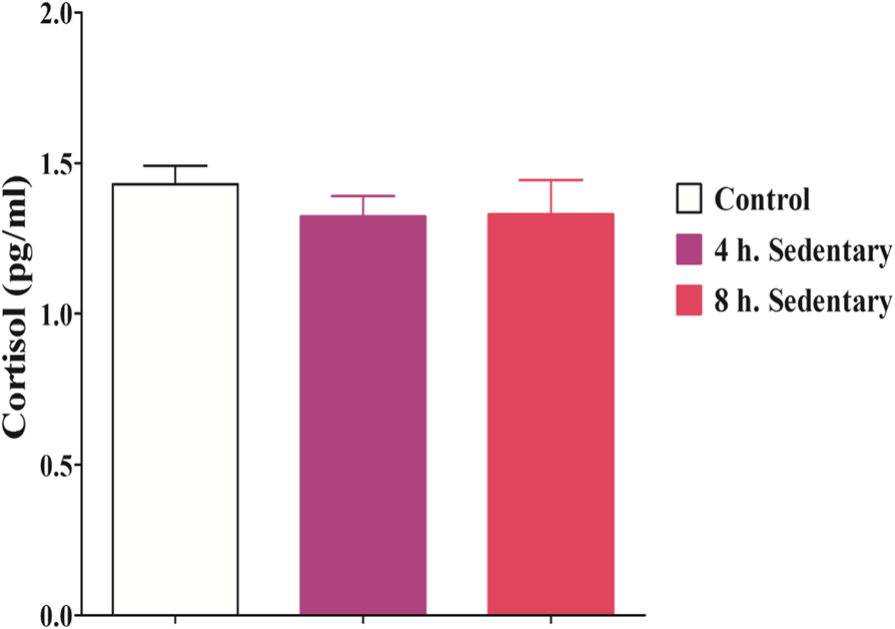
Effect of 3 months sedentariness on cortisol. Each value represents Mean ± SD (*n* = 6)

**Fig. 3:**
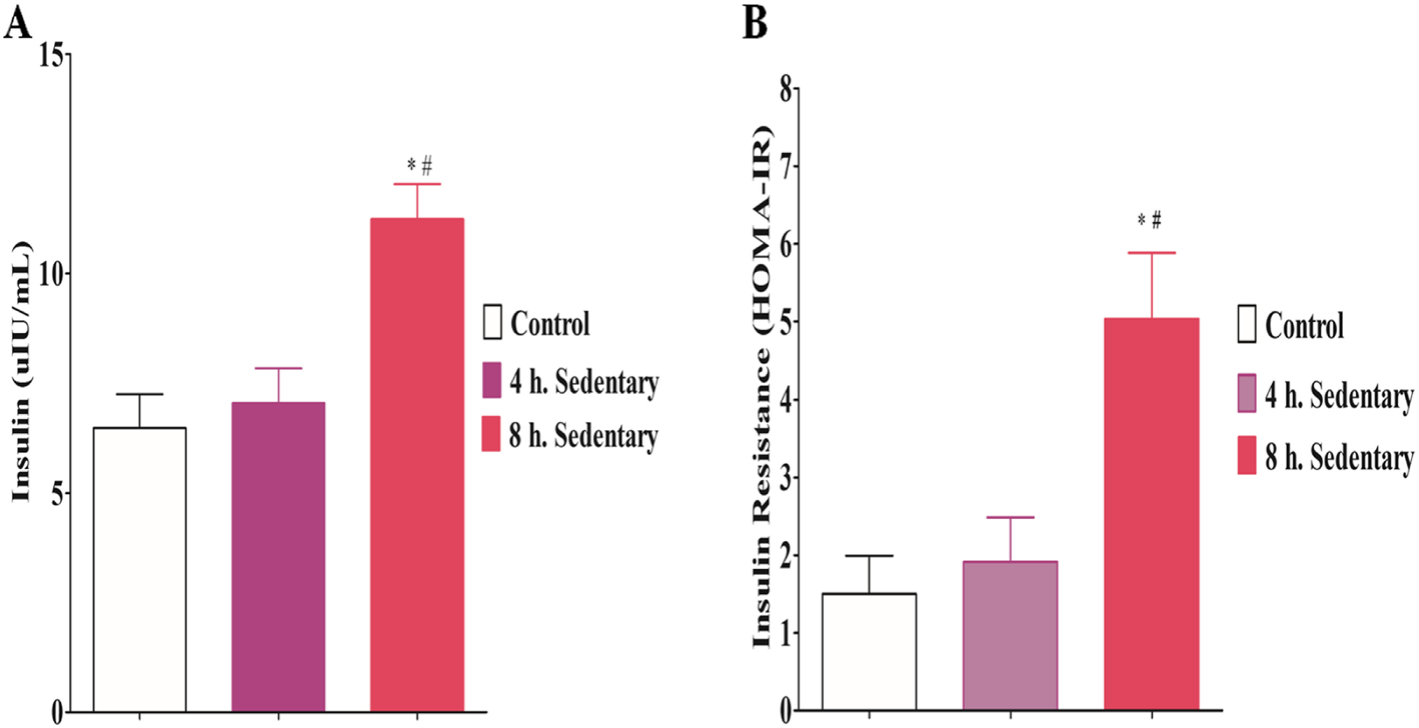
Effect of 3 months sedentariness on serum insulin and insulin resistance (HOMA-IR). Each value represents Mean ± SD (*n* = 6). * = significantly different from control (*p* < 0.05); # = significantly different from 4 h. SED (*p* < 0.05)

There was no significant difference (F = 0.4971; *p* = 0.6180) in serum cortisol level amongst all the groups of rats used in this study (Fig. 2). There was significantly higher serum insulin level (F = 11.02; *p* = 0.0011) in the 8 h. sedentary group when compared with the control and 4 h. sedentary groups (Fig. 3A). The serum insulin resistance of the 8 h sedentary group was significantly higher (F = 51.93; *p* < 0.0001) when compared with the control and 4-h. sedentary group. The higher insulin resistance in this group indicated low insulin sensitivity by the target tissues or reduced responsiveness of target tissues to insulin (Fig. 3B).

### Serum TNF-α, IL-6 and CRP and endothelin-1

**Fig. 4:**
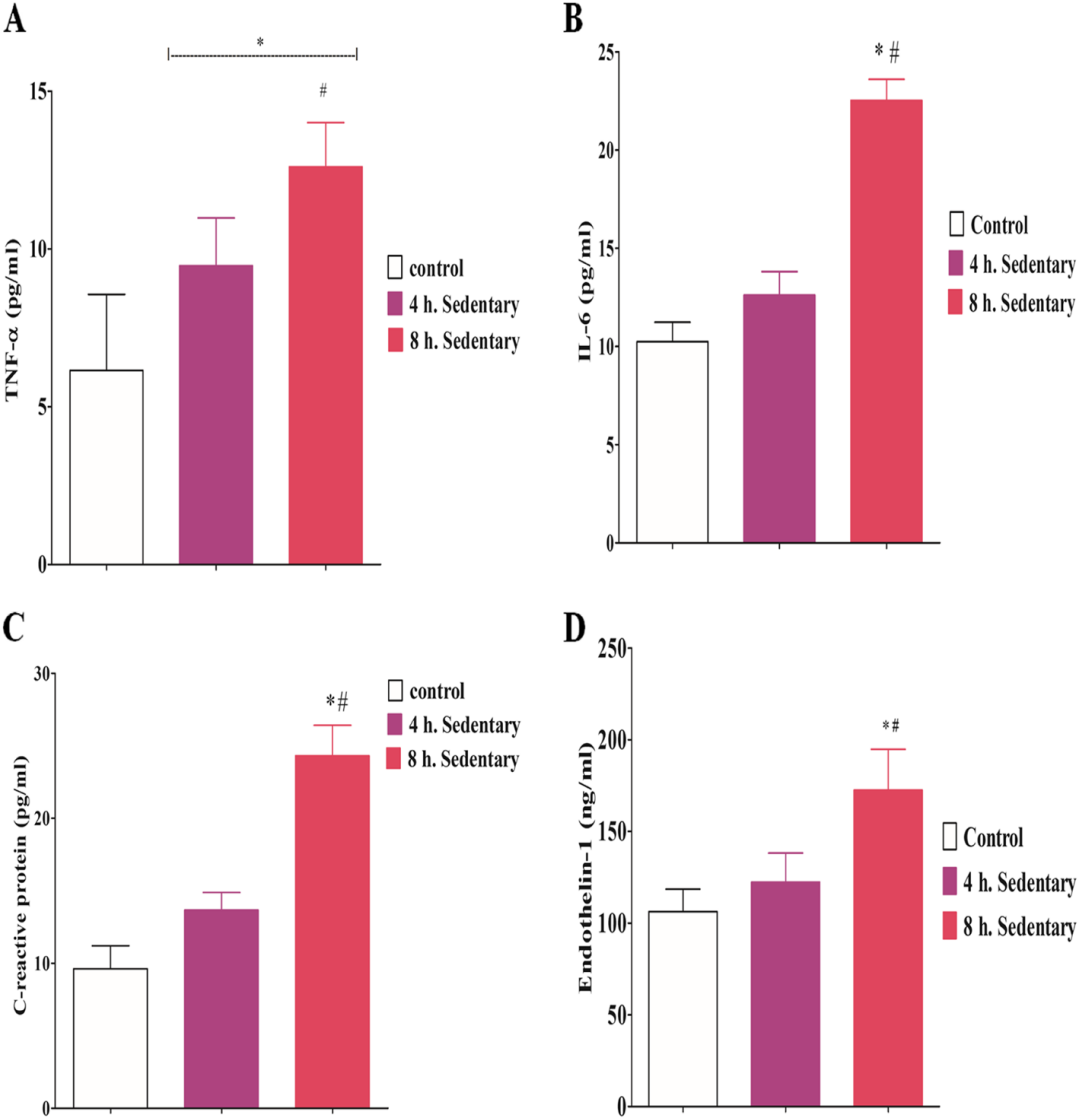
Effect of 3 months sedentariness on tumor necrosis alpha, interleukin 6, C-reactive protein and endothelin-1. Each value represents Mean ± SD (*n* = 6). * = significantly different from control (*p* < 0.05); # = significantly different from 4 h. SED (*p* < 0.05)

TNF-α was significantly higher (F = 18.63; *p* < 0.0001) in the serum of the two sedentary groups when compared with the control (Fig. 4A). Also, 8-h. sedentary group had a significantly higher IL-6 (F = 35.71; *p* < 0.0001), CRP (F = 20.58; *p* < 0.0001) and endothelin-1 (F = 37.75; *p* < 0.0001) when compared with the control and 4 h. sedentary groups (Fig. 4B, C & D). This significantly higher endothelin-1 level in the 8 h. sedentary group signified endothelium dysfunction and vascular constriction (Fig. 4D).

### Correlation between the BMI and biomarkers of cardiometabolic risk, stress and inflammatory process of the non-sedentary and sedentary rats

The correlation analysis revealed association between the BMI and the markers of cardiometabolic risk (glucose, insulin, HOMA-IR, TC, TG, LDL, HDL, atherogenic index, systolic blood pressure, diastolic blood pressure, NO, endothelin-1, albumin-creatinine ratio) and inflammatory process (TNF-α, IL-6 and CRP) in the sedentary rats while there was no strong association between BMI of sedentary with cortisol and uric acid (Table 5).

**Table 5:** Association between BMI, cardiovascular parameters, and inflammatory process parameters of non-sedentary and sedentary rats

	Non-sedentary			Sedentary		
	R^**2**^	r	***P***-value	R^**2**^	r	***p***-value
Glucose (mg/dl)	0.1158	0.3403	0.5092	0.8750	0.9354	< 0.0001***
Insulin (uIU/mL)	0.1484	0.3852	0.4508	0.5206	0.7215	0.0081**
HOMA-IR	0.1946	0.4411	0.3813	0.8130	0.9017	< 0.0001***
TC (mg/dL)	0.1132	−0.3365	0.5144	0.7318	0.8555	0.0004***
TG (mg/dL)	0.2867	0.5354	0.2736	0.7358	0.8578	0.0004***
LDL (mg/dL)	0.0016	−0.0406	0.9392	0.5072	0.7122	0.0094**
HDL (mg/dL)	0.0210	−0.1449	0.7841	0.3821	−0.6181	0.0322*
Atherogenic index	0.3186	0.5645	0.2432	0.6844	0.8273	0.0009***
Systolic Blood Pressure (mmHg)	0.05658	−0.2379	0.6499	0.3421	0.5849	0.0457*
Diastolic Blood Pressure (mmHg)	0.03280	−0.1811	0.7313	0.3341	0.5780	0.0490*
Endothelin-1 (ng/mL)	0.05027	−0.2242	0.6693	0.5621	0.7497	0.0050**
NO (mmol nitrite/mg protein)	0.2026	0.4501	0.3704	0.6212	−0.7882	0.0023**
Albumin-to-Creatinine ratio (mg/g)	0.3825	0.6185	0.1906	0.4407	0.6638	0.0186*
TNF-α (ρg/mL)	0.0359	0.1896	0.1906	0.5005	0.7074	0.0101*
IL-6 (ρg/mL)	9.5060	−0.0098	0.9854	0.6747	0.8214	0.0011**
C-reactive protein (ρg/mL)	0.7224	0.6499	0.1321	0.6491	0.8057	0.0016**
Cortisol (ρg/mL)	0.06192	−0.2488	0.6344	0.0020	0.04497	0.8896
Uric Acid (mg/dL)	0.4836	0.6954	0.1250	0.3247	0.5698	0.0531

Pearsons Correlation (2 tailed)

* = significant at *p* < 0.05

## Discussion

This study designed a novel sedentary cage with a view to achieving sedentariness in rats, and also investigated the effectiveness of cage in achieving sedentariness by assessing some biomarkers of cardiometabolic health in Wistar rats subjected to two different periods of sedentariness in the cage for a period of 3 months. The selected markers were chosen because they have been reported to be significantly altered by sedentary lifestyle in human population studies.

Anthropometric indices such as body weight, BMI and Lee index were measured across all the groups. Increases in these parameters have been reported as indicator of obesity [22, 33]. Previous study reported that BMI and Lee index of rats above 0.68 g/cm^2^ and 0.31, respectively indicated obesity in the rats [22]. The result of the present study revealed that the BMI (0.82 g/cm^2^) and Lee index (0.35) of rats subjected to 8-h. sedentariness were significantly higher compared with the control and 4-h. sedentary groups, indicating an appreciable sedentariness-induced obesity. Moreover, food consumption was significantly increased in 8-h. sedentary group compared with the other groups. Since the energy expenditure of the sedentary rats was minimal, the obvious weight gain could be linked to an imbalance between calorie intakes and energy expended. In this regard, the food intake of the 8-h. sedentary group exceeded their expenditure, which was the basis of weight gain. This result is in congruent with previous studies where sedentary behavior was found to be associated with body weight gain [34–36]. This study indicated that increased food consumption in sedentariness caused significant body weight gain in the rats, as reflected by the higher BMI and Lee index observed in the 8-h. sedentary group.

The results of this study also showed that serum glucose was significantly higher in the 8-h. sedentary group when compared with the other groups. This could be due to a decreased responsiveness of the target tissues of the animals in this group to insulin action (insulin resistance). Also, the serum insulin level and homeostatic assessment of insulin resistance (HOMA-IR) were significantly higher in the 8-h. sedentary group compared to the control and 4-hr. sedentary group. These results affirmed the reduced responsiveness of the target tissues to the insulin action in this group of rats, which could occur as result of the excessive adipose tissue in their target organs. Insulin resistance impaired glucose uptake in the insulin-sensitive tissues such as skeletal muscle, fat and heart, thus disrupting glucose homeostasis [37]. It is believed that insulin resistance leads to hyperinsulinemia because of excessive production of insulin by β-islets in an effort to control the blood glucose. Also, many cases of insulin resistance have been linked with obesity [38, 39]. As a result of energy surplus in cells, the adipose tissue of the rats in the 8-h. sedentary group could mediate ATP release in the mitochondria, subsequently inhibiting adenosine monophosphate-activated protein kinase (AMPK) signaling pathway and thereby blocking the action of insulin and glucose uptake by the target cells [40, 41]. Similarly, the adipose tissue of the sedentary rats could release pro-inflammatory cytokines such as TNF-α and IL-6 as a compensatory mechanism to act on and/or prevent insulin receptor substrate-1 (IRS-1) for the decrease of adipocytes and skeletal muscle insulin sensitivity through the inhibition of serine phosphorylation [42–44].

In the current investigation, the 8-h. sedentary group exhibited significantly higher serum TNF-α and IL-6 when compared with the control and 4-h. sedentary groups. TNF-α expression has previously been reported to increase in the adipose tissues of rats and humans [44, 45]. It was believed to induce insulin resistance in adipose tissues by altering the normal insulin signaling pathway, stimulating adipocyte lipolysis, inhibiting serine phosphorylation of IRS-1 activity thereby decreasing glucose transporter ‘GLUT4’ synthesis and membrane translocation [43–45]. Like TNF, IL-6 level was significantly higher in sedentary rats, particularly the 8-h. sedentary group, when compared with the control. IL-6 has been found to trigger hypertriglyceridemia through the stimulation of lipolysis and hepatic triglyceride secretion in sedentary animals and humans [44, 46]. C-reactive protein (CRP) is synthesized by the liver in response to elevated circulating IL-6 or other factors released by macrophages and adipocytes. IL-6 together with TNF-α act on the liver to stimulate the acute phase response leading to an increase in CRP production and other bioactive mediators [47]. Altogether, these induce insulin resistance by promoting serine-phosphorylation of IRS-1, which impairs insulin signaling and subsequently inhibits glucose uptake by target cells. The population-based cohort and meta-analysis studies showed that inactivity or obesity stimulates hepatic secretion of CRP, which increases the risk of diabetes [46, 48]. These findings support the hypothesis that CRP along with TNF-α, IL-6 is etiologically involved in the pathogenesis of diabetes.

Moreover, elevated serum levels of TNF-α, IL-6 and CRP have been shown to increase the risk of cardiovascular diseases [48, 49]. The results of this study, especially in the 8 h. sedentary group is in line with previous studies, which suggested that patients with elevated levels of CRP and pro-inflammatory cytokines such as TNF-α, IL-6 are at increased risk of diabetes, atherosclerosis, hypertension and other cardiovascular diseases [48, 49].

The result of the lipid profile in the sedentary groups indicated a significantly higher serum total cholesterol (TC), triglyceride (TG), and low-density lipoprotein cholesterol (LDL-C) when compared with the control group. However, the high-density lipoprotein cholesterol (HDL-C) level of the 8-h. sedentary rats was significantly lower when compared with the control and 4-h. sedentary groups. An imbalance in lipid metabolism has been linked with diabetes and insulin resistance [38]. The excessive increase in free fatty acids in the blood results in an elevated use of lipids by tissues, and hence glucose metabolism alteration [50]. There was significant weight gain and elevation of pro-inflammatory markers, IL-6 and TNF, in the serum of the 8-h. sedentary rats. These must have interrupted insulin action on their tissues, reduced lipoprotein lipase and facilitated peripheral tissues lipolysis [44, 51], which undoubtedly hastened dyslipidaemia and hyperglycemia in the sedentary rats. Atherogenic index (AI) is an indicator of lipid metabolism impairment and predictor of cardiovascular disease risk [52]. The significantly raised AI and decreased HDL-C observed in the 8-h. sedentary group suggested the possibility of cardiovascular disease risk in the rats that spent more time in the sedentary cage. These assertions are consistent with the previous report that related sedentary behavior in humans with dyslipidaemia and cardiovascular disease risk [51, 53].

In this study, uric acid and microalbuminuria levels were significantly higher in the 8-h. sedentary rats. The increased serum uric acid level has been reported to be an independent factor for hypoalbuminaemia and microalbuminuria in prehypertensive rats and human subjects [54]. Microalbuminuria is identified as a useful marker of cardiovascular disease risk [55, 56]. Raised levels of these biomarkers is an indication of endothelial dysfunction [57]. It is believed that endothelial dysfunction could be a possible pathway linking uric acid and microalbuminuria with cardiovascular diseases [58, 59]. Hyperuricemia has been reported to inhibit endothelial NO bioavailability [58]. This assertion is buttressed by the significant decrease in serum NO observed in the 8-h. sedentary rats. NO is a potent vasodilatory chemokine [60] majorly found in the vascular system. There is emerging evidence to demonstrate its relevance in vascular biology and increased organ perfusion. However, its significant decrease in the serum of the 8-h. sedentary rats may indicated vascular endothelial dysfunction and subsequently reduced organ perfusion. This affirmation was bolstered by the marker of endothelium dysfunction, endothelin-1. Endothelin-1 is a potent vasoconstrictor that is involved in the implication of hypertension and atherosclerosis [61]. Its significant increase in the 8 h. sedentary rats indicated probable endothelium dysfunction and hypertension.

Moreover, systolic, diastolic and mean arterial pressures were significantly high in the 8-hr. sedentary rats, an indication of high blood pressure. The recorded elevated blood pressure was also confirmed by a significant decrease in NO bioavailability and increase in endothelin-1 in rats subjected to sedentary. The reduced serum NO bioavailability and upsurge in endothelin-1 have been positively linked with hypertension [62, 63]. Also, the dyslipidemia, insulin resistance, microalbuminuria and elevated production of pro-inflammatory cytokines observed in this study, increased the risk of hypertension in the rats subjected to 8-h. sedentariness.

The study also assessed the serum cortisol levels in both control and rats kept in the novel sedentary cage. Although, there were slightly lower serum cortisol levels in the sedentary groups, it was not significant when compared with the control group. This indicated that the sedentary cage did not introduce other stressors that were secondary to their being kept in the cage.

The limitation of this study is that it was not conducted in the nighttime when the animals are mostly more active. However, studies have shown that Wistar rats easily adapt to environmental conditions to which they are subjected [64]. In this study, the rats actually adapted to daytime activities. They were usually awake the daytime because they were always expecting their food and water in the daylight hours. However, studying them at nighttime or inside laboratories with carefully regulated light and dark cycle can be explored in another study.

**Fig. 5:**
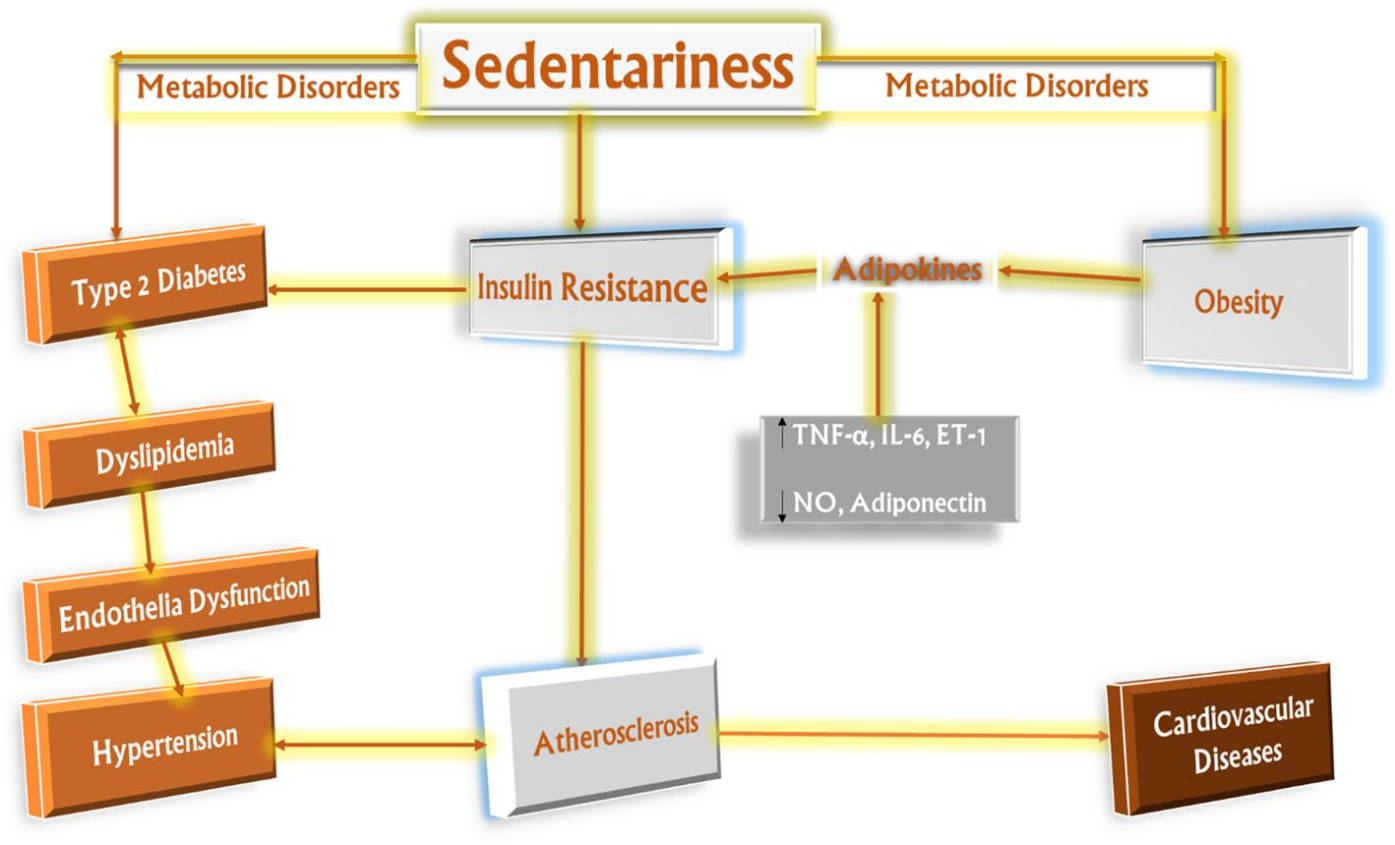
Schematic illustrations of effect of sedentariness on cardiometabolic health. Effects of sedentariness on metabolic syndrome of obesity and insulin resistance on endothelial dysfunction and increased hypertension and atherosclerosis risk. Insulin resistance and adaptive hyperinsulinemia are apparent to be the basis of endothelial dysfunction by promoting endothelial activation and a proatherogenic environment. The adipokines enhance the detrimental effects

In conclusion, this study demonstrated that the novel sedentary cage successfully caused sedentariness in male Wistar rats kept in them, as revealed by significant alterations in the cardiometabolic health (Fig. 5) of the rats, especially the group that were made sedentary for 8 h.

## Data Availability

All data generated and analyzed during this study are presented in the main text, tables and figures. An alternative data format is available from the corresponding author on reasonable request.
